# Biogeochemical behavior and pollution control of arsenic in mining areas: A review

**DOI:** 10.3389/fmicb.2023.1043024

**Published:** 2023-03-23

**Authors:** Fan Zhuang, Jingyi Huang, Hongguang Li, Xing Peng, Ling Xia, Lei Zhou, Teng Zhang, Zhenghua Liu, Qiang He, Feng Luo, Huaqun Yin, Delong Meng

**Affiliations:** ^1^Key Laboratory of Biometallurgy Ministry of Education, School of Minerals Processing and Bioengineering, Central South University, Changsha, China; ^2^Chenzhou Tobacco Company of Hunan Province, Chenzhou, China; ^3^Hunan Renhe Environment Co., Ltd., Changsha, China; ^4^Hubei Key Laboratory of Mineral Resources Processing and Environment, Wuhan University of Technology, Wuhan, Hubei, China; ^5^Beijing Research Institute of Chemical Engineering and Metallurgy, Beijing, China; ^6^Department of Civil and Environmental Engineering, University of Tennessee, Knoxville, Knoxville, TN, United States; ^7^School of Computing, Clemson University, Clemson, SC, United States

**Keywords:** arsenic pollution, bio-geochemical processes, microbial communities, pollution control, mining area

## Abstract

Arsenic (As) is one of the most toxic metalloids that possess many forms. As is constantly migrating from abandoned mining area to the surrounding environment in both oxidation and reducing conditions, threatening human health and ecological safety. The biogeochemical reaction of As included oxidation, reduction, methylation, and demethylation, which is closely associated with microbial metabolisms. The study of the geochemical behavior of arsenic in mining areas and the microbial remediation of arsenic pollution have great potential and are hot spots for the prevention and remediation of arsenic pollution. In this study, we review the distribution and migration of arsenic in the mining area, focus on the geochemical cycle of arsenic under the action of microorganisms, and summarize the factors influencing the biogeochemical cycle of arsenic, and strategies for arsenic pollution in mining areas are also discussed. Finally, the problems of the risk control strategies and the future development direction are prospected.

## 1. Introduction

Arsenic (As) is a toxic metalloid element. Although arsenic As is not a metal, its toxicity is similar to that of heavy metal elements [such as antimony (Sb), cadmium (Cd), chromium (Cr), and lead (Pb)], therefore, arsenic is usually included when talking about heavy metal poisoning. These toxic heavy metals or metalloids are released from a large number of waste residues (mining waste rocks, tailings, and smelting slag) produced in the process of ore mining, beneficiation, and smelting. The released toxic elements further migrate to the surrounding environment under the action of surface runoff, rain, and snow infiltration, resulting in serious environmental pollution. Arsenic is one of the most toxic heavy metals and is classified as the Group 1 human carcinogen by the International Agency for Research on Cancer (IARC) (Zhou and Xi, 2018). Many areas are chronically contaminated with arsenic worldwide. For example, studies have shown that more than half of the Mississippi River Basin is at high risk of arsenic contamination (Yang et al., 2014).

Mining activity is one of the major factors leading to arsenic pollution. Arsenic is the 20th most abundant element in the earth's crust, and most of the As intercalate in gold, copper, lead, zinc, tin, nickel, or cobalt minerals in the form of sulfide (Mandal and Suzuki, 2002). Deposition of mining waste including mine rock, mine tailing, and smelting slags leads to serious heavy metal pollution in the surroundings, and arsenic is the main pollutant causing ecological risk to the environment around the mining area. The migration, transformation, and enrichment of arsenic in the soil–rice system are higher than those of other heavy metal elements such as lead and zinc. It threatens human health and ecological security.

In this article, the distribution and migration of arsenic, the geochemical cycle of arsenic under the action of microorganisms, the factors influencing the biogeochemical cycle of arsenic in the mining area, and the strategies for pollution control are summarized and prospected.

## 2. Distribution and migration of arsenic in the mining area

Heavy metal pollution around the mining area mainly originates from the oxidation and dissolution of sulfide ore. Weathering and leaching of abandoned rocks, mine tailings, and smelting slags that are produced during mining activity lead to As being released and dispersed into the surrounding environment (Okkenhaug et al., 2012). Therefore, mining waste containing As is the main source of arsenic contamination. Oxidation of sulfur will generate an acidic solution. Heavy metal ions in smelting slag, waste ore, and mine tailings diffuse into the surroundings through leaching effects (e.g., chemical acid leaching or microbial leaching) (Yan et al., 2017). Arseno-bearing sulfide (e.g., FeAsS) in the mine waste can be oxidized to form scorodite (i.e., FeAsO_4_). Except for sulfur oxidation leading to acid solubilization of As, arsenic can also be dissolved under reducing conditions. Studies have shown that a heap of tailings and slags in the mining area would cause reducing conditions, and iron oxide or iron hydroxide would be reductively dissolved, resulting in a large number of As in tailings and slag release into environments (Al-Abed et al., 2007). Both As and antimony (Sb) can be released under reduction conditions. As and Sb are homotopes, with similar chemical characteristics. In the mining areas, As is reported to have the highest environmental risk (Xue et al., 2023). As and Sb mainly occur in anion forms (i.e., arsenate and arsenite or antimonate and antimonite), which is different from other cation heavy metals, such as Cd, Pb, Zn, or Cu. Generally, in mining wastes with both Sb and As, Sb would be first released or whose release is stronger than As release, and thus, As would release after Sb. This may be due to the reason that the metabolism of Sb (both oxidation and reduction) may compete with As for electron acceptors or donors (Bagherifam et al., 2019) and lead to differences in As metabolism between As mining sites and other heavy metal contaminated environments. It is reported that the distribution of the arsenic content in different areas of mining varied from 70 to 5330 mg/kg (Otones et al., 2011), and the vertical distribution of As through soil profiles suggests a deposition mechanism of this element on the topsoils that involves both biotic and abiotic factors (Yang et al., 2020).

The chemical form and occurrence speciation of As would change through a series of biochemical reactions, where chemical forms and speciation determine the toxicity and bioavailability of arsenic. According to Tessier's sequential extraction method, heavy metals existed as five fractions, including exchangeable fraction, carbonate-bounded fraction, Fe/Mn oxide-bounded fraction, organic matter-bounded fraction, and residue fraction (Tessier et al., 1979). The speciation of heavy metals determines their solubility, mobility (Weng et al., 2002), and bioavailability (Zimmerman and Weindorf, 2010). It is reported that in soils, arsenic mainly existed in residual fraction, followed by organic matter-bounded fraction, Fe/Mn oxide-bounded fraction, carbonate-bounded fraction, and exchangeable fraction; among the fractions, residue state constituted 25–50%, and iron-manganese oxide bond state constituted 21 to 35% (Zhao et al., 2022). Even though the distribution pattern of fractions in mining sites differed between studies, the residual fraction was the main component (i.e., Yang et al., 2020; Zhao et al., 2022). In mining water, the main forms of arsenic are arsenate and arsenite. In the lower pH range (1 to 3), the main chemical forms of arsenate As (V) in acid mine drainage (AMD) were H_3_AsO_4_ and H_2_${\text{AsO}}_{4}^{-}$, while arsenite As (III) mainly exists in the form of H_3_AsO_3_. Yet at typical pH (4 to 9) for most surface and ground waters, As(V) is present as a negatively charged oxygen anion (e.g., H_2_${\text{AsO}}_{4}^{-}$ or ${\text{HAsO}}_{4}^{2-}$), while As(III) is present in the neutral (H_3_AsO_3_) form (Cheng et al., 2009). Published research on the distribution and migration of heavy metals in mining areas and their environmental risks was mainly based on the change in the occurrence forms and total amounts (Yang et al., 2020), while the distribution of As chemical forms (i.e., valence state) that highly determines toxicity was rarely reported. Different from some heavy metals (e.g., Pb and Cd) that mainly exist in inorganic forms, As can exist in both inorganic and organic forms. Inorganic arsenic has two valence states: As (III) (such as arsenite) and As (V) (such as arsenate). As (III) has strong water solubility and high mobility, while As (V) has relatively weak water solubility and low mobility, but this does not mean that As (V) is difficult to be bio-absorbed. For example, rice seedlings effectively absorb both As (V) and As (III) under liquid culture conditions. Under aerobic conditions, soil Fe (III) adsorbs As (V) and then decreases the bioavailability of As. Organic arsenic mainly includes methyl arsenide, sulfur-containing methyl arsenide, and chlorine-containing methyl arsenide. Methylated arsenic, on the one hand, can be microbial demethylated to produce inorganic arsenic, and on the other hand, it is further converted to volatile arsenyl hydrogen compounds [monomethylarsenic hydride (MMAsH2), dimethylarsenic hydride (DMAsH), and trimethylarsenic (TMAs)] (Yan et al., 2015). At present, it has also been found that in addition to TMAs, there are methyl arsenic chloride gas and methyl arsenic sulfur gas in the arsenic gas released by the geothermal environment (Planer-Friedrich et al., 2006). The biological effectiveness and toxicity of different forms of arsenic vary. Generally for living organisms, inorganic arsenic is significantly more toxic than organic arsenic. Dimethyl arsenates DMAs(V) and trimethylarsenic oxides (TMAsO) are significantly less toxic than As (III) (Cui et al., 2014), but toxicity increases substantially when DMAs (V) and TMAsO are reduced to dimethyl arsenites DMAs(III) and trimethylarsenic TMAs (III) (TMAs) (Styblo et al., 2000). Therefore, the environmental risk and health toxicity of arsenic can be reduced to some extent when inorganic arsenic in the environment or in living organisms is converted to less toxic organic arsenic or volatilized into the atmosphere as a gas. Since As in the same occurrence form can contain a variety of chemical forms with different toxicity (such as arsenite and arsenate can coexist in the exchange state), it is more in-depth and accurate to study the distribution, migration, and ecological risk of As pollution from the perspective of chemical valence states.

## 3. Microorganisms are the important driving force for the geochemical cycle of arsenic

Soil microbes and heavy metals can interact with each other. The diversity, abundance, and function of microorganisms can be significantly affected by heavy metals. With the increase in heavy metal concentration, the aggregation of the microbial community is more decisive. Heavy metal stress makes the prokaryotic community deterministic, but its effects on the assembly process of different microbes are different (Zhang Y. et al., 2020). The microbial community composition, as well as network interactions, was shifted to strengthen the adaptability of microorganisms to heavy metal contamination (Li et al., 2017). In addition, bacteria showed different reactions to heavy metals. For example, *Anaerobic microbes*, such as *Anaerolineaceae*, not only play important roles in shaping the microbial community in soils but also might be involved in regulating Cd solubility (Meng et al., 2019). *Chlorella* can biomineralize Pb under the promotion of montmorillonite to photosynthesis and urea hydrolysis (Tan et al., 2022). On the contrary, microorganisms are the core driving forces that lead to the transformation of different forms of arsenic.

**Figure 1:**
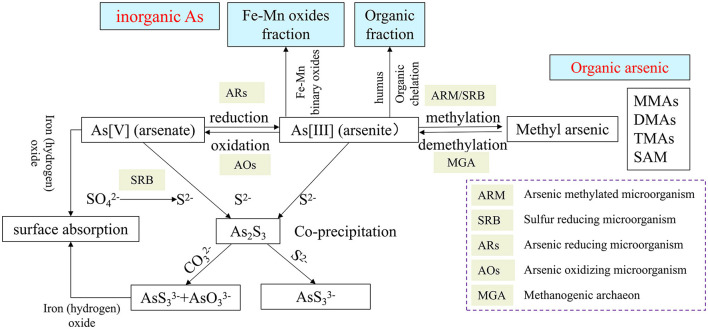
Biogeochemical processes of arsenic and functional microorganisms involved.

The transformation behavior of As (i.e., the geochemical behavior of arsenic) includes oxidation–reduction, methylation and demethylation, organic chelation, surface adsorption and dissociation, and ion co-precipitation. Of these, arsenic reduction, demethylation of organic arsenic, and dissociation of adsorbed arsenic form highly toxic As(III), leading to increased arsenic mobility and toxicity, while arsenic oxidation, methylation, organic chelation, and co-precipitation produce less toxic/mobile arsenates, volatile methyl arsenic (e.g., DMAs and TMAs), organically bound arsenic, and residual arsenic sulfide, reducing arsenic contamination (Bianco Prevot et al., 2018). Many geochemical processes of As involve the participation of microorganisms (Figure 1). Various direct or indirect As metabolic activities of microorganisms are the main driving forces for the geochemical behavior of As (Barrera-Diaz et al., 2012; Meng et al., 2019). Microorganisms can directly carry out arsenic reduction, arsenic oxidation, methylation, and demethylation and can also be indirectly involved in the oxidation of arsenic and ion co-precipitation. It should be mentioned that many microorganisms can conduct biological volatilization of arsenic to convert arsenic compounds into volatile derivatives, such as methylation of arsenic. Bio-volatilization of arsenic is a new subject in biogeochemistry and environmental health, which plays an important role in the global biogeochemical cycle of As and can also be used as a potential arsenic bioremediation method (Wang et al., 2014). Almost all microorganisms have been reported to possess an As resistance and metabolism gene (Zhu et al., 2017).

There are specific genes driving the As metabolism, including reduction, oxidation, methylation, or demethylation (Table 1). The genes and microorganisms containing As metabolism genes were widely detected in mine slag, fresh water, sediments, soil, hot spring, marine water, and other samples. Li et al. (2019) reported that microorganisms in the mining environments (i.e., acid mine drainage) can derive As metabolism genes such as *arsA, arsR*, and *arsB* through horizontal gene transfer (HGT), suggesting those genes may not have species specificity. Some others also reported HGT events of As metabolism genes, but the genes can also be vertically transferred (VGT) (Dunivin et al., 2019). As these processes dramatically alter the toxicity and bio-efficacy of arsenic, the study of microbial arsenic metabolism genes is important for understanding environmental arsenic metabolic processes and microbial remediation potential. In mining areas, the increase in arsenic concentration and the activation of arsenic in bedrock aquifers are caused by several geochemical processes, including bedrock weathering, oxidation of arsenopyrite and main sulfides in ore, the mixture of mine water and surface water, leaching arsenic alkali residue, and adsorption–desorption from iron/manganese oxide/hydroxide (Wen et al., 2018). Inorganic As is converted into organic As by methylation of soil microorganisms and volatilizes into the atmosphere, thus releasing and migrating As in gaseous forms (Bentley et al., 2002; Turpeinen et al., 2002). Volatile arsenic-containing compounds can be oxidized in the atmosphere and then enter into the soil or water with rain or atmospheric dry deposition, finally completing the circulation of As in the soil/water body and the atmosphere.

**Table 1:** Major genes involved in arsenic metabolism.

	**Species specificity**	**Gene**	**Faction**	**References**
Reduction of As(V)	Mainly on plasmids of gram-negative bacteria such as *Escherichia coli*.	*arsRDABC*	The arsR gene encodes a trans-repressor protein of the ArsR/SmtB family to regulate the transcription of the operon. arsB encodes an AS III efflux protein, while arsA and arsD are responsible for encoding two additional proteins, ARSA, an ATP enzyme activated by AS III, and ArsD, a transcription repressor protein responsive to another metal. Ars primarily confers detoxification of inorganic arsenic compounds.	Patel et al., 2007; Yan et al., 2019
	Mainly on plasmids or chromosomes of gram-positive bacteria such as *staphylococcus aureus*. Eukaryotic organisms such as Trichoderma asperellum SM-12F1, Penicillium janthinellum SM-12F4, and Fusarium oxysporum CZ-8F1	*arsRBC*		
	*Chrysiogenes arsenatis Shewanella sp* Members in phylum Proteobacteria	*arrA*	encodes the As reductase	
Oxidation of As(III)	Members in phyla Proteobacteria, Actinobacteria, Firmicutes and Bacteroidetes	aioA/B	Exists in autotrophic arsenic oxidizing microorganisms (CAO) that utilize As(III) oxidation energy supply for chemotaxis (Aerobic)	Sultana et al., 2012; Shi et al., 2020
		*aox*	Exists in arsenic oxidizing microorganisms (HAOs) that simply oxidize and detoxify to improve arsenic resistance.	
	Proteobacteria and Eurycota	*arxA*	As (III) oxidase ArxA can only work together with the reduction of NO${}_{3}^{-}$ under chemoautotrophic conditions. (anaerobic)	Yan et al., 2019
Organic As oxidation	*Pseudomonas putida*	*arsH*	Oxidize organic MAs(III) to MAs(V)	Chen et al., 2015
	*Paracoccussp. SY Escherichia coli*	*arsV*	Oxidize methylarsenite	Chen J. et al., 2021
Methylation and demethylation	*Rhodopseudomonas*	*arsM*	often adjacent to other genes encoding arsenic detoxification proteins.	Ye et al., 2012
	*Bacillius* MD1	*ArsI* *(ArsI C-As lyase)*	Demethylation of MAs(III) to As(III)	Yoshinaga and Rosen, 2014
Absorption and efflux of As (III)	*Saccharomyces cerevisiae*	*acr*	As (III) Efflux in fungi	Bhattacharjee and Rosen, 2007; Tsai et al., 2009
		*arsB* *arsA*	Arsenate efflux in bacteria	
	*Schizosaccharomyces*	*fps*	Is a bidirectional channel protein that transports As(III)	Ghosh et al., 1999
	*Campylobacter jejuni*	*arsP*	Efflux of trivalent methylarsenicals [MAs(III)]	Yan et al., 2019
	*Escherichia coli*	*arsW*	Extrude methylarsenate	Chen J. et al., 2021
	*Ensifer adhaerens* ST2	*arsK*	Methylarsenite efflux gene	Chen J. et al., 2021

### 3.1. As reduction

The two mechanisms of microbial reduction of elemental arsenic include dissimilatory arsenic reduction and cytoplasmic arsenic reduction. Dissimilatory arsenic reduction refers to the reduction of As (V) to As (III) by microorganisms using compounds such as lactate and acetate as electron donors, and these microorganisms are known as dissimilatory arsenic-reducing prokaryotes (DARPs, dissimilatory arsenate-reducing prokaryotes) (Lukasz et al., 2014). More than 30 species of DARPs have been found, distributed in the genera *Shewanella, Halomonas*, and *Bacillus*. DARPs possess *arrAB* operon, which can be activated and expressed by As (V), and encode dissimilatory arsenic reductase to catalyze the reduction reaction of As (V) (Amend et al., 2014) [to form As (III)]. The reduction of cytoplasmic arsenic is catalyzed by a series of enzymes encoded by *ars* operons and carried out in the cytoplasm of microorganisms, where As (V) is reduced to As (III) with higher activity and then pump out of the cells by membrane carrier proteins encoded by *arsB*, thus achieving arsenic detoxification (Rosen, 2002). In addition, the As reduction has also been found in eukaryotic organisms such as *Trichoderma asperellum* SM-12F1, *Penicillium janthinellum* SM-12F4, and *Fusarium oxysporum* CZ-8F1 (Su et al., 2015).

### 3.2. As oxidation

As(III) can be oxidized to produce As(V) by arsenic-oxidizing microorganisms, under both aerobic and anaerobic conditions using either O_2_ or ${\text{NO}}_{3}^{-}$, respectively, as electron acceptor(s). The arsenic-oxidizing microbes include chemoautotrophic arsenic-oxidizing microorganisms (CAOs) and heterotrophic arsenic-oxidizing microorganisms (HAOs). CAOs can use oxygen as an electron acceptor to oxidize As (III) to produce As (V) catalyzed by arsenic oxidase enzyme that contains two subunits, a large subunit aioA (with molybdopterin and a [3Fe-4S] cluster) and smaller subunit aioB (with a Rieske-type [2Fe-2S] cluster). Another type of As(III) oxidase, arxA, was also identified in *Alkalilimnicola ehrlichii* strain MLHE-1 (Zargar et al., 2010). It was found that the *arxA* can only work together with the reduction of ${\text{NO}}_{3}^{-}$ (Yan et al., 2019). HAOs can utilize arsenic oxidase in peripheral cytoplasm to catalyze As (III) to produce As (V) (Yan et al., 2019; Shi et al., 2020). The reported arsenic oxidation functional microorganisms include *Rhizobium* and *Thermus aquaticus*, and they were widely found in arsenic-contaminated soil, sediment, and water (Yamamura and Amachi, 2014). Furthermore, it reported the identification of the *nemRA* manipulator extracted from Enterobacteriaceae, which was shown to be involved in the oxidation of trivalent organic arsenic and to be regulated by the trivalent organic arsenic selective transcriptional repressor *NemR* (Shi et al., 2021). In addition, the organic methyl arsenite can be oxidized to methyl arsenate by the methyl arsenite-specific oxidase arsH or arsV and then be extruded by arsW, arsP, or arsK to increase microbial resistance to organic As (Chen et al., 2015; Chen J. et al., 2021).

### 3.3. As methylation

Microbial arsenic methylation generates volatile organic arsenic, which drives the biogeochemical cycle of arsenic from inorganic to organic. Biogenesis of arsenic methylation is widespread in nature, and arsenic methylation gene (*arsM*) is widely distributed in the genome of different species. Studies have shown that a*rsM* orthologous protein is distributed in bacteria, archaea, and eukaryotes, including Cyanobacteria, Bacteroidetes, Firmicutes, and Proteobacteria (Zhao et al., 2013). Trimethyl arsenic is the main product of microbial methylated arsenic. It is shown that sulfate-reducing bacteria (SRB) could participate in the methylation process of inorganic arsenic, and their research results also indicated that methanogenic archaea were involved in the demethylation process of methylated arsenic (Chen et al., 2019).

### 3.4. As demethylation

Compared with microbial arsenic methylation, there are very limited studies associated with As demethylation in microbial cells. Yoshinaga and Rosen (2014) reported that *Bacillus* stain MD1 can use ArsI to demethylate MAs(III) to form As(III). *Mycobacterium* can demethylate monomethyl arsenite [MMAs (V)] or monomethyl arsenite [MMAs (III)] into a mixture of arsenite [As (V)] and arsenite [As (III)] (Lehr et al., 2003). In addition, the mixed cultures of *Burkholderia* and *Streptomyces* can be reduced and demethylated. It is proved that the demethylation of MMA (V) to As (III) is a two-step process (Yoshinaga et al., 2011). Moreover, some methylating microorganisms can demethylate methylsalicylic acid. Arsenic-methylating fungi, such as *Fusarium oxysporum* CZ-8F1, *Penicillium janthinellum* SM-12F4, and *Trichoderma asperellum* SM-12F1, can demethylate dimethyl arsonic acid [DMA (V)] to As (V) and As (III) (Su et al., 2015).

### 3.5. As bioleaching and co-precipitation

Microorganisms can also indirectly participate in the biogeochemical cycling of arsenic. Iron–sulfur-oxidizing microorganisms form acidic environments by oxidizing iron and sulfur elements in ores, resulting in the release of arsenic from ores, which are the main sources of arsenic pollution in mines (Wu et al., 2021). Microbial-mediated redox of iron has an important influence on the environmental behavior of arsenic, such as dissolution and release of arsenic, adsorption and precipitation, and morphological transformation. The extracellular electron transfer process of iron oxidation bacteria promotes the mineral phase transformation of iron and couples with arsenic passivation (Zhao et al., 2022). The product of manganese-oxidizing microorganisms, the biogenic manganese oxides, can oxidize As (III) to As (V), and microbial manganese oxidase (*CumA*) inhibits the expression activity of *arsC* gene of arsenic-reducing bacteria (Akhtar et al., 2013). Dissimilatory iron-reducing bacteria (DIRB), such as *Geobacter* and *Shewanella*, can reduce trivalent iron in iron (hydroxide) oxides to divalent iron, resulting in the deletion of arsenic adsorption sites and the release of adsorbed arsenic to activate metal ions in the solid phase (Liu et al., 2020). *Geobacter* can use acetic acid as a carbon source and electron donor to drive the reduction process of As (V) (Wang et al., 2016). Sulfate-reducing bacteria (SRBs) such as *Desulfuromonas* reduce sulfate to produce sulfide, which co-precipitates with trivalent arsenic in the soil to form insoluble arsenic sulfide, immobilizing free heavy metal ions (Sun et al., 2020). For instance, *Klebsiella* sp participated in the phosphate co-precipitation of Cr, and the highest removal rate of Cr reached 95% under soil conditions (Gupta et al., 2018). In the pH range of 3–12, the fixation rates of Cd, Pb, and Zn in soil by phosphate co-precipitation are 20–97.9%, 62.3–99.9%, and 28.6–98.7%, respectively (Chen et al., 1997). Phosphate-solubilizing microorganisms can solubilize phosphorus into phosphate that can co-precipitate with heavy metals to form arsenic phosphate minerals (Jiang et al., 2019).

## 4. Factors affecting arsenic biogeochemical cycle and risk control strategies in the mining area

Human activities, climatic conditions, geological background, and soil properties can directly or indirectly affect the biogeochemical cycling of arsenic through the action of microbial communities.

Soil physical (mineralogical characteristics, particle size, porosity, and climatic conditions) and chemical (temperature, pH, potential, and organic matter) (Abbasi et al., 2021) properties would affect the adsorption characteristics of heavy metals and, therefore, play important roles in regulating the geochemical behavior of arsenic (Burton et al., 2020). Soil oxidation–reduction potential (EH) has a strong effect on the migration and speciation of As in abandoned soil. Arsenic mobilization increases at moderately reducing conditions, while it reduces under oxic conditions (Mensah et al., 2022a). As can aggregate with the oxides and hydroxides of Fe resulting in bioavailability and mobility reduction (Bai et al., 2022). Therefore, As pollution and migration are affected by the existence of iron and manganese oxide and hydroxide adsorption (Wen et al., 2018). In addition, the rate (percentage) of soluble As in acidic soil is generally higher than that in alkaline soil. Carboxyl, phenol, and alcohol groups of soil organic matter can aggregate with metal ions to form complexes through inner cohesion and surface adsorption. As ions that are embedded in mineral lattice structures or stably complexed with complex organic matters are insoluble/insoluble in natural conditions, thereby affecting As mobility and toxicity (Egli et al., 2010).

Carbon, nitrogen, and sulfur compound can react with heavy metals or serve as electron donors or acceptors and, therefore, have a significant impact on As behavior. Sulfur element facilitates the conversion of iron oxide-bound arsenic to sulfide minerals, thereby enhancing its stability (Chen et al., 2022). In soil, phosphorus, particularly inorganic P, can release soil-retained As (mostly arsenate) by competing for adsorption sites (Wu et al., 2022). The addition of sodium sulfate and elemental sulfur reduced the mobility of As in the soil solution and the concentration of As (III), thus reducing the toxicity of As. Sulfate mainly affects the reduction process of sulfur and iron in soil and further affects the mobility and form of As in soil (Yan et al., 2022). Nitrogen is one of the most important basic elements in the biosphere, and different forms of nitrogen have different effects on the migration and transformation of As in soil. The addition of nitrate nitrogen can reduce the concentration of As in pore water in a short time, and the addition of high concentration ammonium nitrogen can reduce the concentration of As in the whole culture period (Liu et al., 2022). By promoting the reduction of Eh and inhibiting the reduction of dissimilated iron and the transformation of arsenic species, nitrate can be used as an effective modifier for the immobilization of As in soil (Chen Z. et al., 2021).

Human activities in the mining area such as smelting, waste/tailings management, and pollution control measures (such as pollution isolation, solidification stabilization, and phytoremediation) can directly or indirectly affect As distribution, migration, and toxicity (Wang et al., 2017; Ye et al., 2018). Studies showed that mining, dressing, and smelting activities in the mine area had the highest contribution rate of 46.6% to As pollution in the river sediments in the lower reaches of the mine area (Zhang et al., 2018). Therefore, it is necessary to clarify these questions:

(1) How do human activities affect the core driving force (microorganism) of the arsenic biogeochemical cycle? Human factors mainly include a large amount of arsenic released by human activities, which directly or indirectly enters the environment. The mining of mineral activities, the piling and leaching of coal gangue, and the discharge of bottom ash and fly ash from coal burning in mining areas will cause arsenic pollution to different degrees in the soil around the mining area. As we all know, the increase in arsenic concentration in the soil directly affects the activity of the soil microbial community. There is a negative correlation between the number of soil microbial communities and arsenic concentration. The chemical forms of arsenic in soil determine the transformation, migration, and toxicity of arsenic to a great extent, and the activities of soil microbial communities in turn affect the formation of arsenic and its compounds, so it may be possible to reduce arsenic pollution in mining areas by adjusting the structure of microbial communities (Simon, 2000). Studies have shown that the dominant phylum of soil biota is Proteus, Cyanobacteria, Actinomycetes, and Bacteroides, which play a key role in the formation of soil microbial communities of *aioA* and *arsM*. Soil organic carbon (OC), pH, and chlorophyll a (Chl a) are the most important environmental factors to change soil microbial communities, respectively, while it had a significant negative correlation with available As, As(III), and total As (Mao et al., 2023).

(2) How are the prevention and control approaches related to soil properties, microbial functions, As distribution and migration, and ecological toxicity? Biogeochemical cycling of As driven by microorganisms will affect the occurrence and chemical forms of As in ore areas, resulting in the migration and transformation of As and changes in ecological toxicity, such as pollution in the mining area and the surrounding environment. Previous studies on the migration, transformation, and ecological toxicity of As in the mining environment often neglected the key role of microorganisms in the biogeochemical cycle of As, which limited the research results to the immediate state of As pollution and lacked prediction on the changing trend of As pollution. As a result, the evaluation of As pollution remediation strategies lacked efficiency and predictability. Therefore, in-depth study on microbial diversity, composition, and function (such as *ars* operator, *arrAB* operator, *cumA, sor, dirb*, and other genes or functions) in the arsenic-contaminated environment of the mining area is needed. In combination with the distribution and migration law of arsenic element, scientific prediction is made on the changing trend of arsenic form and migration law in mining area environment, so as to provide a theoretical basis for the assessment and control of arsenic pollution risk in the mining area.

(3) What are the key factors that dominate As distribution and migration in mining areas, so as to provide theoretical basis and technical guidance for arsenic pollution prevention and control in areas with different climatic conditions, geological backgrounds, and soil properties? The forms of arsenic in the environment are changeable, so it is a research hot spot to study the changes and migration of arsenic in the environment. PH value is the primary factor to control the formation and transformation of secondary minerals. Studying the long-term stability of arsenic-containing secondary minerals in the mine environment and controlling arsenic migration behavior may be the key to improving the efficiency of arsenic pollution control and governance in future.

The control and prevention approaches for toxic metal elements in mining areas include pollution isolation technology (control), solidification and stabilization technology (resistance), and *in situ* reduction technology (reduction) (Table 2).

**Table 2:** Control and prevention approaches for toxic metal elements.

**The control and prevention approaches for toxic metal elements**	**Advantages**	**Disadvantages**
Pollution isolation technology (control)	Effectively control the migration and diffusion of toxic elements into the surrounding soil, water and other media.	1. Poor control effect on vertical migration of pollution elements. 2. The contradiction between short-term effect and long-term service performance of isolation barrier is difficult to coordinate.
Solidification and stabilization technology (resistance)	1. Short processing time. 2. wide application range.	1. Affected by the complex mine environment, material stability and environmental compatibility. 2. Low efficiency of stabilization and repair. 3. Short duration.
*In-situ* reduction technology (reduction)	1. Effectively maintain water and soil of mine. No damage to soil ecological environment. 2. Safety and environmental protection. 3. Relatively low cost.	1. Large required area. 2. Time consuming.

*Pollution isolation technology* can effectively control the migration and diffusion of toxic elements into the surrounding soil, water, and other media. Except for a series of construction technologies that have been developed, such as cutoff slurry walls using mainly thin walls, cement–bentonite–water slurries, sheet piles walls, jet grouting curtains, injection walls, and bored-pile cutoff walls, artificial ground freezing has been applied to pollution control in recent years (Anekwe and Isa, 2021; Rajendran et al., 2022). In this process, the pore water in the soil pores freezes and fills the gaps, which reduces the permeability of the soil and prevents the diffusion and transfer of pollutants (Bello et al., 2018). However, this technology mainly controls the horizontal lateral transfer of the pollution elements and has a poor control effect on the vertical migration of the pollution elements, and the contradiction between the short-term effect of the isolation barrier and the long-term service performance is difficult to coordinate.

*Curing stabilization technology* converts the soluble state of heavy metals in mine soils into insoluble states through the addition of passivating materials such as minerals, micro-nano, microorganisms, and chelator (Chen et al., 2009) that undergo characteristic adsorption, organic chelation and biomineralization, and chemical immobilization, physical adsorption, and physical encapsulation *in situ* to reduce the activity and mobility of contaminants and their diffusion in the mine. For instance, some microorganisms like cyanobacteria have a good remediation effect on arsenic in mining soil. Experiments show that the total As(T) and available As(a) in tailings soil decreased by 12.73 and 27.65%, respectively, after cyanobacteria inoculation (Qi et al., 2023). In addition to microorganisms, many nanomaterials can also be used to stabilize arsenic. Biochar-loaded nanoscale zero-valent iron (nZVI@BC) was prepared for remediation of arsenic-contaminated soil, which exhibited the best immobilization performance, significantly promoted the transformation from labile arsenic to iron–aluminum oxide-binding state (Song et al., 2022). It is noteworthy that there are quite a few examples of successfully using such methods. As an example, the use of citric acid and rhamnose as chelating agents has reportedly resulted in the removal of 83.65% of arsenic from contaminated soil around an abandoned smelter in China (Ke et al., 2020). Another example is that amorphous manganese oxide (AMO) was used as an amendment to chemically immobilize heavy metal-contaminated soil from a Czech lead smelter, resulting in a 52.64% reduction in reactive arsenic in the soil (Ettler et al., 2015). However, the strength of the reaction between the immobilized materials and heavy metal ions and the duration of action are affected by the mine's complex environment (climate, soil properties, and microorganisms), material stability, and environmental compatibility. The unclear geochemical behavior, biological driving mechanism, and environmental action principle of the toxic metal elements are the key bottlenecks that lead to the low efficiency and short duration of solidification and stabilization remediation. In addition, the lack of comprehensive and systematic data research in the mining area is also an important factor limiting the efficiency of solidification and stabilization restoration.

*The in situ reduction technology* is mainly based on the super heavy metal accumulation plants that can tolerate heavy metal toxicity and can extract heavy metals, which reduces the total amount of heavy metal elements in the mine environment. The plant roots can effectively maintain the water and soil in the mine to reduce the migration and diffusion of heavy metal elements into the surrounding environment. However, large polluted biomass needs to be treated properly. It still lacks green and efficient methods for treating polluted biomass. Plant root exudates can affect the speciation, migration, transformation, and ecological toxicity of metal elements in the environment. Moreover, it can change soil physical and chemical properties (organic matter content, water content, Eh, pH, and available nutrient content) and microbial community structure and function and indirectly affect microbial-mediated geochemical behavior of metal elements. The complex mechanisms remain to clarify further. At present, many studies and cases have proved the practicability of this method. For example, ryegrass can adsorb arsenic, and the use of manure alone and in combination with compost could improve the remediation efficiency of ryegrass on arsenic-contaminated soil (Mensah et al., 2022b). At the same time, many researchers have developed innovative methods to combine metal-tolerant plants with microbial inoculation and *in situ* immobilization as a means of phytoremediation (Xu et al., 2021).

The effectiveness of control and prevention measures for As or other toxic metal elements in mining areas can usually be assessed by measuring the bioavailability, fractional change and total change of As. In addition, risk assessment, including ecological risk assessment and health risk assessment, is also used to evaluate the effectiveness of control and prevention approaches. Other than accessing As in the remediated soil, the underground water is also used for assessment interventions (Xue et al., 2023). Chemical stabilization techniques reduce the risk of migration and diffusion of heavy metals in the environment by applying stabilizing materials to convert them from a highly reactive state to a less reactive state (Palansooriya et al., 2020) and are one of the main technical tools for risk management of heavy metal-contaminated soils, which are fast, economical, and efficient. In the practice of stabilization of heavy metal-contaminated soils, different leaching methods can be used, such as the AA method (using nitric sulfate), TCLP method (using acetic acid) (Cui et al., 2018), and DTPA method (using CaCl2-TEA-DTPA slow flush solution) (Zhang et al., 2022), which are used to determine the changes in the concentration of heavy metals in the active state of the soil before and after stabilization. The continuous grading method classifies heavy metals in soil into different active forms according to the extraction order, assesses the remediation effect of heavy metal stabilization in contaminated soil by the changes in the distribution of different heavy metal forms, and then investigates the stabilization mechanism (Zhang H. et al., 2020). The choice of specific solutions needs to be based on the remediation technology developed in different stages, scenarios, and soil heavy metal pollution control objectives required.

## 5. Conclusion and perspectives

The remediation of heavy metals such as As pollution will inevitably cause changes in biological and non-biological properties in the mining ecological system, in which the arsenic metabolism/transformation microorganisms will mediate the biogeochemical cycle process of arsenic, and finally affect the migration and transformation of arsenic and ecological toxicity. However, how do As metabolism/transformation microorganisms drive biogeochemical cycling of arsenic in mining areas? How can the key action factors of remediation approaches affect the migration and transformation of arsenic element and ecological toxicity? Such scientific problems are still unclear, which leads to the blindness in the formulation of control for heavy metal pollution in mining areas and the unsatisfactory effect of technical prevention and control.

At present, many studies developed a variety of new methods for simultaneous extraction and detection of arsenic chemical forms (Sun et al., 2015; Zhang et al., 2021), and the contribution of ore activities to arsenic pollution in the surrounding environment (farmland soil and sediment) was analyzed (Zhang et al., 2018), elucidating the biotoxicity and bioaccumulation process of arsenic with different chemical forms (Song et al., 2020; Cui et al., 2021), revealing the arsenic tolerance of arsenic-oxidizing microorganisms and arsenic oxidation mechanism (Li et al., 2019). On the basis of the aforementioned research, in future, one can use high-performance liquid chromatography–plasma mass spectrometry (HPLC-ICP-MS), *in situ* synchrotron radiation techniques (μ-XRF and μ-XANES), multi-omics analysis techniques, and system dynamics models to analyze the biogeochemical cycle process and microbial driving mechanism of arsenic by studying the occurrence/chemical morphological characteristics, distribution, and migration law of arsenic in mining areas, together with molecular bioinformatic tools, to make clear the key factors that affect the migration and transformation of arsenic and ecological toxicity and to reveal the internal mechanism of pollution control strategies to control arsenic pollution. The simulated arsenic pollution model mining area needs to be constructed, which should cover geological background, human activities, soil properties, arsenic metabolizing microorganisms, arsenic biogeochemical cycle process, arsenic mobility, and diffusion degree. A forward-looking arsenic pollution prevention and control strategies are expected to be put forward to provide a theoretical basis and technical guidance for the efficient prevention and control of arsenic pollution in the mining area.

## Author contributions

FZ, JH, and DM wrote the manuscript. All authors helped in editing and completing the manuscript. All authors contributed to the article and approved the submitted version.
